# Polarization of THP-1-Derived Human M0 to M1 Macrophages Exposed to Flavored E-Liquids

**DOI:** 10.3390/toxics13060451

**Published:** 2025-05-29

**Authors:** Raivat Shah, Emily D. Luo, Carly A. Shaffer, Maya Tabakha, Sophie Tomov, Siara H. Minton, Mikaela K. Brown, Dominic L. Palazzolo, Giancarlo A. Cuadra

**Affiliations:** 1 Department of Biology, Muhlenberg College, 2400 W. Chew Street, Allentown, PA 18104, USA; raivat@med.umich.edu (R.S.); emilyluo@upenn.edu (E.D.L.); cs1964@pcom.edu (C.A.S.); mtabakha@muhlenberg.edu (M.T.); stomov@muhlenberg.edu (S.T.); 2 University of Michigan Medical School, 1500 E. Medical Center Drive, Ann Arbor, MI 48109, USA; 3 University of Pennsylvania School of Dental Medicine, 240 S 40th St, Philadelphia, PA 19104, USA; 4 Philadelphia College of Osteopathic Medicine, 4170 City Ave, Philadelphia, PA 19131, USA; 5 DeBusk College of Osteopathic Medicine, Lincoln Memorial University, Harrogate, TN 37752, USA; siara.minton@lmunet.edu (S.H.M.); mikaela.brown@lmunet.edu (M.K.B.); domenico.palazzolo@lmunet.edu (D.L.P.)

**Keywords:** E-liquids, THP-1 cells, M0 and M1 macrophages, viability, MHC class II, CD80, cytokines

## Abstract

Electronic cigarettes (ECIGs) are widely used but their effects on the immune system need to be further investigated. Macrophages are white blood cells central to the immune response. Using THP-1-derived M0 macrophages, this study aims to determine the effects of ECIG liquids (E-liquids) on the polarization of M0 to the pro-inflammatory M1 macrophage subtype. THP-1 cells were cultured and differentiated to M0 macrophages using RPMI media. E-liquids ± cinnamon, menthol, strawberry and tobacco flavors were added to cell cultures at 1% (*v*/*v*) during polarization with lipopolysaccharides and interferon γ for 24 to 72 h. Morphology, viability, gene expression and cytokine production were measured using light microscopy, the LDH cytotoxicity assay, qPCR and ELISA, respectively. The results show that cells present little to no LDH activity under any treatments. In addition, cinnamon-flavored E-liquid severely affects morphology (i.e., abolishing pseudopodia formation), gene expression of all genes tested, and cytokine production. Other E-liquid flavors also affect some of these parameters, but to a lesser extent. Our data suggest that E-liquids can affect the polarization from M0 to M1, thus affecting the immune response in ECIG-exposed tissues such as the mucosa in the oral cavity and airways, ultimately mitigating the health status.

## 1. Introduction

The use of electronic cigarettes (ECIGs) among youths and adults has exponentially surged over the past decade to epidemic proportions [1,2,3]. Sales of ECIGs reached USD 28.18 billion in 2023 and is expected to grow by 30.6% by 2030 [4]. In a period of just ten years (2013 to 2023), the global use of ECIGs increased over 300%, from 25.4 to 86.1 million [5]. Such statistics have increased concerns about the health implications of vaping, necessitating further research. The dramatic increase in vaping, especially among young adults, is particularly alarming [6]. Although ECIG products are available in many forms, they are generally composed of a rechargeable battery, a compartment containing the ECIG liquid (E-liquid), and a heating element capable of vaporizing the E-liquids [7]. E-liquids are typically composed of propylene glycol (PG) and/or vegetable glycerin (VG) as the base humectants, nicotine in varying concentrations, and a myriad of appealing flavoring additives [8].

In the current cultural milieu, many consider vaping to be safer than traditional smoking, since vaping is free of the harmful ingredients typically generated from combusted tobacco [9]. In addition, this belief in harm reduction is often promoted as a tobacco cessation tool, and to circumvent the negative tobacco-related effects present in traditional cigarette smoking [10]. On the other hand, commercially available E-liquids typically contain concentrations of nicotine that are higher than conventional cigarettes, contributing to the highly addictive potential of ECIGs [11]. Furthermore, E-liquids and their generated aerosols also contain hazardous materials, such as organic compounds, ultrafine particles, heavy metals such as nickel, tin, and lead, and possibly other cancer-causing chemicals [12,13,14,15,16]. Perhaps more concerning is the many flavor additives, which appeal to young adults and have been shown to induce extensive pathological effects on many tissues [17,18,19,20,21].

The scientific literature contains several investigations showing hazardous effects of flavoring agents using in vitro and ex vivo models. A recent study published by our group shows that oral epithelial cells display deficits in mucin production, wound-healing capabilities and levels of glutathione, as well as an increase in interleukin (IL)-8 [22]. Additionally, osteoblasts treated with cinnamon-flavored E-liquid and its aerosol decrease in viability and increase in oxidative stress [23]. Furthermore, menthol-flavored E-liquid has a harmful effect on human periodontal ligament fibroblasts reducing migration, ATP production and cell viability [24]. An ex vivo study using 6- to 8-week-old C57BL/6 mice evaluated changes in nervous, cardiopulmonary and digestive tissues after 3 months of daily flavored aerosol exposure (20 min three times a day) and their results suggest that chronic inhalation of ECIG can lead to inflammatory changes across multiple organ systems [25]. In contrast, few studies focus on the differentiation and function of macrophages as these cells are central to the immune system.

Blood monocytes, derived from hematopoietic stem cells in the bone marrow, circulate until infection or inflammation necessitates monocyte recruitment into nearly any resident tissue, at which point differentiation to macrophages occurs [26,27]. Uniquely, macrophages display high functional plasticity, enabling specific shifts in phenotype in response to stimuli. These phenotypic shifts include the activation of M1 macrophages in response to pro-inflammatory stimuli, and the alternative M2 macrophages under anti-inflammatory conditions, both arising from the naive M0 precursor [28,29,30,31]. Classical activation of M1 macrophages can take place during injury, infection, or inflammation in vivo or during in vitro experimentation by exposure to bacterial cell wall constituents such as lipopolysaccharide (LPS) as well as pro-inflammatory cytokines such as interferon (IFN)-γ, tumor necrosis factor (TNF)-α, and granulocyte-monocyte colony-stimulating factor (GM-CSF) [30,32]. Once activated, M1 macrophages are characterized by the secretion of pro-inflammatory cytokines such as TNF-α, IL-1β, IL-6 and IL-12. In addition, M1 macrophages upregulate the expression of major histocompatibility complex (MHC) class II, which present antigen peptides to T cells, as well as cluster of differentiation (CD) 80 [33], which acts as a costimulatory signal for T cell activation [34,35,36]. Toll-like receptor (TLR)-4 recognizes LPS on Gram-negative bacteria and is upregulated in M1 macrophages to enhance phagocytosis [28,37,38]. Other functions of M1 macrophages include, but are not limited to, secretion of both pro-inflammatory and antimicrobial mediators [39,40] and T-cell antigen presentation [30,41]. It is worth noting that nicotine, a common constituent of E-liquids, can influence M0 polarization towards the M2 phenotype [42]. In addition, nicotine is also known to facilitate platelet activation [43], which could also induce polarization from M0 to M2 macrophages [44], thus contributing to pro-thrombotic as well as anti-inflammatory effects [45]. This could have significant ramifications when considering potential vaping-induced oral pathologies [45].

Cinnamon- and menthol-flavored E-liquids are known to display a significant effect on macrophage biology. For example, exposure of alveolar macrophages to sub-lethal concentrations of ECIG vapor condensates increases reactive oxygen species production and significantly inhibits phagocytosis [46]. Alveolar macrophages exposed to different flavored E-liquids, where cinnamaldehyde is a common constituent, showed impaired macrophage functions [47]. Moreover, Lee et al. (2019) found that ECIG flavorings induce macrophage polarization into a pro-inflammatory state, with the most detrimental effects observed in the presence of cinnamon and menthol flavorings [48]. E-liquids increase pro-inflammatory cytokine release in macrophages [49], as well as cytotoxicity and reactive oxygen species [21]. Despite these investigations, the implications of flavored E-liquids and their aerosols on macrophage differentiation remains largely unexplored.

Therefore, using the Tohoku Hospital Pediatrics-1 (THP-1), a human leukemia monocytic cell line [50], the aim of this study is to evaluate polarization markers from M0 to M1 macrophages in the presence of E-liquids ± flavors. Specifically, morphology, cytotoxicity, gene expression of MHC class II, CD80 and TLR-4, as well as cytokine expression, are explored. We hypothesized that E-liquids and their flavors have the capacity to negatively impact macrophage differentiation. E-liquid-induced alterations in M0 to M1 polarization could affect the inflammatory response in mucosal tissues exposed to these insults, ultimately compromising the health status of the host. For the convenience of the reader, abbreviations used in this study are listed and defined in Table 1.

## 2. Materials and Methods

### 2.1. Reagents and Supplies

Unless otherwise noted, all materials and reagents used in this study were obtained from (Thermo Fisher Scientific, Waltham, MA, USA).

### 2.2. E-Liquids Preparation

To avoid any unknown compounds in the commercially available E-liquids [51], a 1:1 (*v*/*v*) mixture of food-grade PG and VG (Liquid Nicotine Wholesalers, Phoenix, AZ, USA) was used as the base humectant and supplemented with 20 mg/mL (S)-(-)- nicotine (Alpha Aesar, Tewksbury, MA, USA). Concentrated stocks of tobacco, menthol, cinnamon, and strawberry flavors, also purchased online from Liquid Nicotine Wholesalers, were used to yield a final 5% (*v*/*v*) flavored E-liquid mixture. All final E-liquids were stored at 4 °C for up to two months.

### 2.3. THP-1 Culturing, Differentiation, and Polarization

THP-1 cells were procured from Dr. Angela Brown (Lehigh University, Bethlehem, PA, USA) and cultured in Roswell Park Memorial Institute (RPMI) 1640 (Gibco, Billings, MT, USA) media containing 10% Fetal Bovine Serum (FBS), 1:100 penicillin/streptomycin antibiotics under standard conditions (37 °C, 5% CO_2_) as previously described [52]. The cells divided approximately once per day and were routinely passaged twice a week; keeping the concentration between 0.2 and 1.2 × 10^6^ cells/mL in RPMI media. All experiments were performed between passages 10 and 35. THP-1 cells were differentiated and polarized as previously described [52]. Briefly, the cells were seeded at 200,000 cell/mL/well in 24-well plates and differentiated with 200 nM phorbol 12-myristate 13-acetate (PMA) for 48 h at standard conditions. At this point, THP-1 cells ceased to divide and differentiate to M0 macrophages, which were then polarized to M1 macrophages using the M1 cocktail consisting of human recombinant interferon-gamma (hrIFN-γ) and LPS at final concentrations of 20 ng/mL and 100 ng/mL, respectively, for 24 h under standard conditions [52]. Light microscopy confirmed proper differentiation from THP-1 to M0 macrophages, as indicated by round and flat cells adhered to 24-well plate surfaces, and from M0 to M1 macrophages, as indicated by continued adherence and the presence of typical pseudopodia. For light microscopy, images were collected using a Nikon Digital Sight DS-Fi1 camera mounted on a Nikon Eclipse TE2000-U inverted microscope with NIS Elements Imagine Software, version 3.10 (Nikon Instruments Inc., Melvin, NY, USA) at 100× magnification.

### 2.4. THP-1 Growth and Viability with E-Liquids

THP-1 cell growth kinetics were evaluated while exposed to E-liquids ± flavors. THP-1 cells were seeded at 300,000 cells/mL/well in 24-well plates and E-liquids ± flavors were added to the media at 1% (*v*/*v*). The cells were incubated under standard conditions and cell count and viability were determined using the trypan blue exclusion assay according to instructions [53] at 24, 48, 72 and 96 h.

### 2.5. Polarization of M0 to M1 Macrophages with 1% E-Liquids

To study the effects of E-liquids ± flavors on the polarization of M0 to M1 macrophages, THP-1-derived M0 cells were cultured as indicated above and treated with the M1 cocktail (see Section 2.3) spiked with 1% E-liquids ± flavors in the media. The cells were incubated under standards conditions for 24 h and light microscopy was performed to assess M1 cellular morphology at 24, 48 and 72 h post E-liquid treatments. In addition, at matched time points, 100 µL samples of cell culture supernatants were collected and stored at −20 °C for lactate dehydrogenase (LDH) cytotoxicity assays (see Section 2.7).

### 2.6. E-Liquid Interference with LDH Enzymatic Activity

Lactase dehydrogenase is one of the most abundant enzymes in the cytoplasm of mammalian cells. Toxic compounds could induce cell membrane damage, which would result in the accumulation of LDH in the culture supernatant. Therefore, the LDH cytotoxicity assay measures the enzymatic activity of LDH in the supernatant and it is typically employed to evaluate levels of cellular cytotoxicity after treatment with a toxic agent [54]. To assess the potential interference of E-liquid flavors on LDH enzymatic activity, increasing concentrations of flavored E-liquids were added to this assay. Briefly, M1 macrophages treated with the lysis buffer of the LDH assay kit yielded 100% cellular LDH release. These cell lysates were mixed with 1%, 2%, 4%, 8% and 12% flavored E-liquids and the LDH activity assay was performed following the manufacturer’s instructions [55]. Absorbance at 490 nm and 680 nm was measured using a µQuant monochromatic microplate reader (MTX Lab Systems, Bradenton, FL, USA).

### 2.7. LDH Cytotoxicity Assay

The culture supernatant samples collected above (Section 2.5) were used to evaluate cytotoxicity on M1 macrophages using the LDH cytotoxicity assay, as described above (Section 2.6). Briefly, 50 µL supernatant samples were mixed with 50 µL reaction mixture of the LDH assay kit in 96-well plates and allowed to incubate in the dark at room temperature for 30 min. Fifty microliters of the stop reagent (LDH assay kit) was added to terminate the enzymatic reaction. Absorbances of the final assay mixtures were measured as indicated above (Section 2.6).

### 2.8. RNA Extraction, cDNA Reverse Transcription and qPCR

Total RNA was collected from THP-1-derived M0 macrophages as well as polarized M1 macrophages. In addition, total RNA was collected from M1 macrophages polarized with and without E-liquids ± flavors. Briefly, supernatants were removed, and macrophages were washed once with 1 mL PBS. Total RNA was extracted using the RNA Preparation kit (Zymo Research, Irvine, CA, USA) following the manufacturer’s instructions [56]. RNA was reverse-transcribed to cDNA with the VILO reverse transcription kit following the manufacturer’s instructions [57]. The NanoDrop 2000c (Thermo Scientific, Waltham, MA, USA) was used to determine RNA and cDNA concentrations. A real-time amplification of *18S rRNA* (internal control gene), *Human Leukocyte Antigen (HLA) DR*, a genetic isotype that encodes for MHC class II, *CD80*, and *TLR-4* was performed in the StepOnePlus Real-Time PCR machine (Applied Biosystems, Foster City, CA, USA) using TaqMan assays. The conditions for qPCR were as follows: denaturing at 95 °C for 5 s, annealing and extension at 60 °C for 30 s for a total of 40 cycles. Finally, using the Ct values, 2^−ΔΔCt^ analysis was performed to determine the expression levels of the target genes.

### 2.9. Cytokine Production

Supernatants from E-liquid-treated M1 macrophages were stored at −80 °C. Enzyme-linked immunosorbent assays (ELISA) were used to quantify TNFα, IL-1β, IL-6 and IL-8 production. The assays were performed in 96-well plates according to the manufacturer’s instructions [58,59,60,61] and absorbance was determined at wavelengths of 450 nm and 570 nm using a Synergy H1 (Bioteck, Winooski, VT, USA) microplate reader. The results were calculated to ng/mL based on the assay’s standard curve.

### 2.10. Statistical Analysis

For each treatment group in all experiments, the means ± standard error of the means (SEM) or ±standard deviation (SD) were calculated. Statistical significance between treatment groups for all experiments was determined using Student’s *t*-test, One-Way ANOVA or Two-Way ANOVA. The One-Way and Two ANOVAs were followed by Bonferroni post hoc analysis. Differences were considered statistically significant when *p* < 0.05.

## 3. Results

### 3.1. THP-1 Growth and Viability with E-Liquids

**Figure 1:**
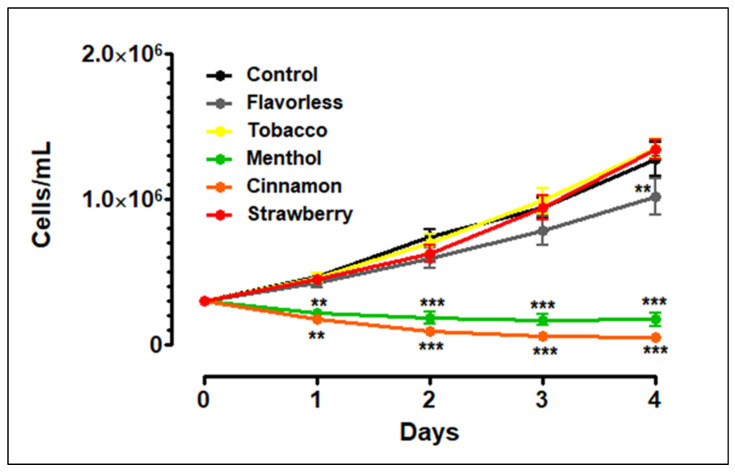
Effects of 1% E-liquids ± flavors on THP-1 cell growth over 4 days. Each point represents mean ± SD; *n* = 4. Statistical significance was determined using a Two-Way ANOVA where ** = *p* < 0.01 and *** = *p* < 0.001 compared to control.

To evaluate the effects of E-liquids ± flavors on THP-1 growth, the cells were initially seeded at 300,000 cells/mL and allowed to grow for four days. Control cells grew in linear fashion from 300,000 to 1,275,000 cells/mL over a four-day period (Figure 1). In comparison to control, the addition of 1% tobacco- or strawberry-flavored E-liquids does not alter THP-1 cell growth. Similarly, the addition of 1% flavorless E-liquid does not alter THP-1 cell growth, except for a slight decrease seen on day four. In contrast, the addition of 1% menthol- or cinnamon-flavored E-liquids significantly decreases THP-1 cell growth to 169,000 and 44,000 cells/mL by day four, respectively. Based on the trypan blue exclusion assay, the data suggest that these flavors are toxic and induce cell death.

### 3.2. Cellular Morphology of M0 to M1 Macrophages in the Absence or Presence of 1% E-Liquids

**Figure 2:**
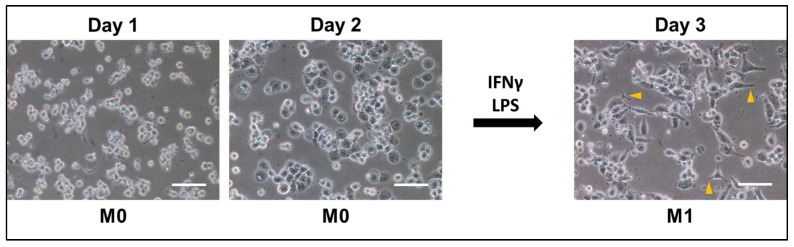
Micrographs of THP-1-derived M0 and M1 macrophages. M0 cells are shown at 24 and 48 h post PMA differentiation (Day 1 and Day 2) and M1 cells are shown 24 post polarization (Day 3). Yellow arrow heads indicate pseudopodia on M1 macrophages. Representative micrographs from five independent experiments were taken at 100× magnification. White bars = 100 µm.

To assess the differentiation of THP-1-derived M0 macrophages and subsequent polarization to M1 macrophages, the cells were treated as previously described (see Section 2.3). Figure 2 shows spherical cells that are well adhered to the surface of the plate by day 1. By day 2, the cells morphed from spherical to a flatter shape but still round. Lastly, after the addition of IFN-γ and LPS, the cells polarized to a typical M1 appearance where pseudopodia of various lengths are clearly visible (yellow arrow heads, day 3), extending from the cells. The results show successful differentiation to M0 and polarization to M1 subtypes.

**Figure 3:**
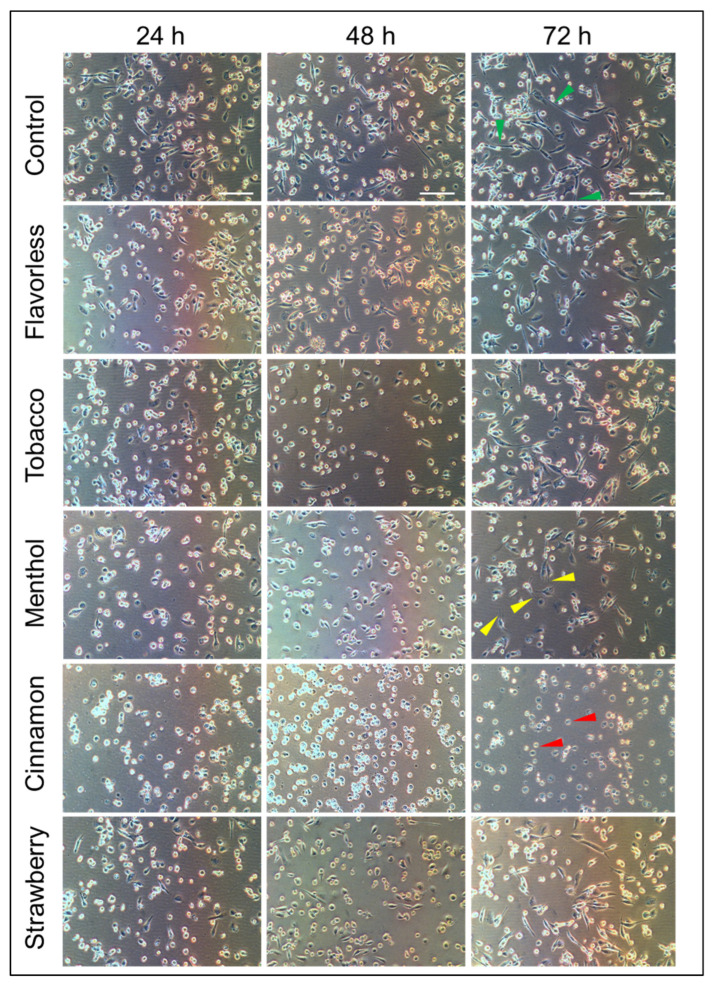
Micrographs of THP-1-derived M1 macrophages polarized with and without 1% E-liquids ± flavors and images taken at 24, 48 and 72 h. Representatives micrographs from four independent experiments were taken at 100× magnification. Green and yellow arrow heads indicate long or short pseudopodia, respectively. Red arrow heads indicate lack of pseudopodia. White bars = 100 µm.

Figure 3 shows the morphology of M0 macrophages polarized to M1 while exposed to 1% E-liquids ± flavors over three days. Cells polarized to M1 without E-liquid treatments show the expected M1 appearance with pseudopodia, as seen in Figure 2. Over time, M1 control cells continue to elongate their pseudopodia, as indicated by the green arrow heads (Figure 3). Exposure to flavorless, tobacco and strawberry E-liquids has little to no effect on the morphology of M1 macrophages, as seen by the presence of pseudopodia (24 h), which continue to elongate over 48 and 72 h. Arguably, the addition of menthol E-liquid seems to slow down the morphological changes as pseudopodia are either shorter or less common (yellow arrow heads). In contrast, the addition of cinnamon-flavored E-liquid, while polarizing M0 to M1, results in macrophages without pseudopodia and cells remain rounded (red arrow heads). Thus, depending on the ECIG flavor, morphological changes, as macrophages are polarized to M1, may be altered over the course of three days. Such alterations could lead to detrimental implications during the immune response.

### 3.3. LDH Cytotoxicity

**Figure 4:**
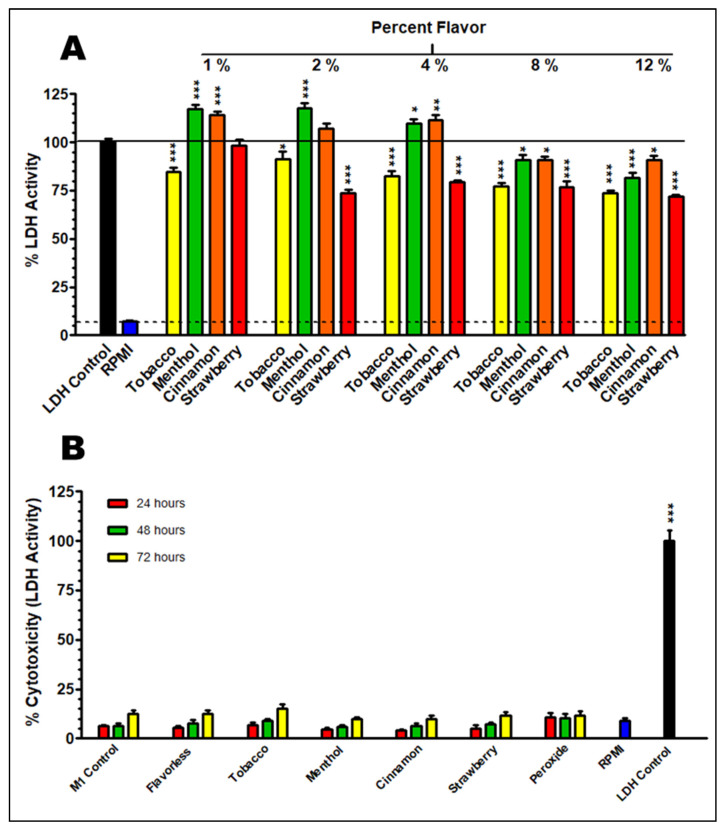
Interference of E-liquids with flavors on THP-1-derived LDH activity. Cell lysates were exposed to 1%, 2%, 4%, 8%, or 12% E-liquid flavors, either tobacco, menthol, cinnamon, or strawberry, and LDH activity is compared to that of LDH control and RPMI. * = *p* < 0.05, ** = *p* < 0.01, *** = *p* < 0.001 compared to 100% activity (**A**). LDH activity on THP-1-derived M1 macrophages with and without 1% E-liquid ± flavors over 24, 48, and 72 h after polarization. LDH activity is compared to control (M1, peroxide, RPMI, and LDH controls) *** = *p* < 0.001 compared to all other groups (**B**). Each bar represents the mean ± SEM; *n* = 6 to 12. Black solid and dashed lines indicate 100% cytotoxicity from LDH activity of lysed THP-1 cells and background LDH activity from RPMI media, respectively.

To evaluate the cytotoxic effects of 1% E-liquid ± flavors over the course of three days, x6cell supernatants were analyzed for LDH activity as an index of membrane integrity. Since the E-liquids are present in the supernatants, we also tested the possible interference of these chemicals on LDH enzymatic activity. Figure 4A shows modest or no interference of E-liquids and their flavors on this biochemical activity. One-percent E-liquid with tobacco decreases LDH activity to 83.3% (*p* < 0.001), and menthol and cinnamon increase it to 119% and 115%, respectively (*p* < 0.001), while strawberry does not show any interference. Because of these variances in LDH activity, the cytotoxicity assay shown in Figure 4B has been mathematically adjusted to reflect these interferences. Nevertheless, all treatments, including 5% peroxide, show LDH activity to be low, ranging from 4.00% to 15.02% when cells are exposed to E-liquid with cinnamon for 24 h, and E-liquid with tobacco for 72 h, respectively. Furthermore, none of these E-liquid treatments yield LDH activity significantly different than that of the M1 control within their respective time points. Overall, these results indicate that treatments with 1% E-liquid ± flavors over the course of M0 to M1 polarization for up to 72 h do not induce cytotoxicity, as measured by LDH activity in the culture supernatants.

### 3.4. Expression of HLA DR, CD80 and TLR-4

**Figure 5:**
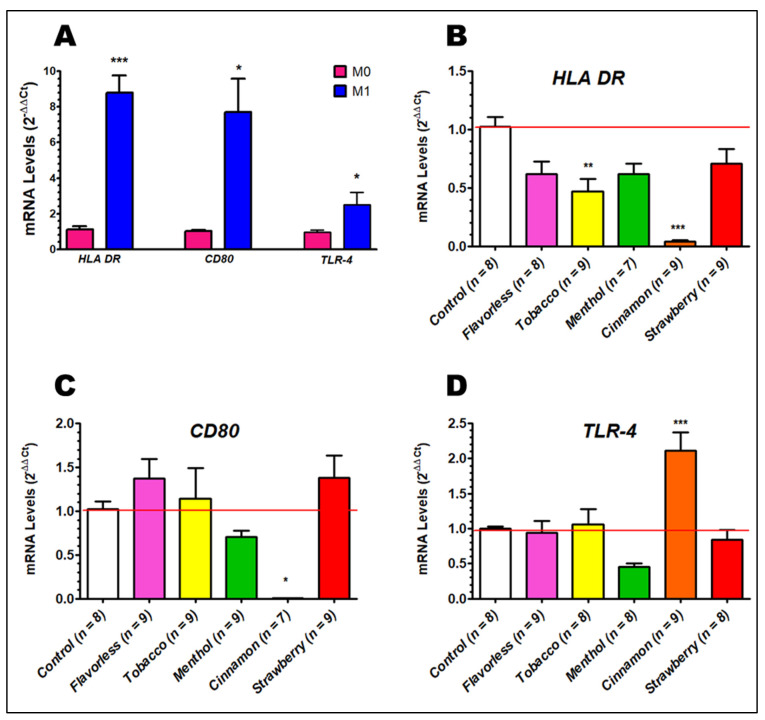
Expression of *HLA DR*, *CD80* and *TLR-4* in M0 and M1 macrophage with and without E-liquids ± flavors determined by qPCR after 24 h of polarization. Gene expression of the three genes in M1 macrophages without E-liquid treatments compared to M0 subtypes (**A**). Expression levels of *HLA DR* (**B**), *CD80* (**C**) and *TLR-4* (**D**) polarizing M0 to M1 macrophages with E-liquids ± flavors compared to untreated controls. Each bar represents the mean ± SEM; *n* = 6 to 12. * = *p* < 0.05, ** = *p* < 0.01, *** = *p* < 0.001. Red lines indicate the control levels of gene expression. All Ct values used fall within 10 and 40.

*HLA DR*, which encodes for MHC class II, *CD80* and *TLR-4* mRNA levels were evaluated on M0 and M1 macrophages prior to treatments with 1% E-liquids. Figure 5A shows the upregulation of all three genes when M0 macrophages are polarized to the M1 subtype without any E-liquid treatments. Expression of these genes significantly increases to 8.8, 7.7 and 2.5 times (*p* < 0.05) for *HLA DR*, *CD80* and *TLR-4*, respectively, over M0 levels. When M0 macrophages are polarized to the M1 subtype in the presence of cinnamon flavored E-liquid, *HLA DR* and *CD80* expression significantly decreases compared to control (*p* < 0.05) (Figure 5B,C), but *TLR-4* expression is significantly elevated under the same treatment (*p* < 0.001) (Figure 5D). In addition, *HLA DR* expression is also affected by tobacco flavored E-liquid (*p* < 0.01). All other treatments do not result in significant changes. Overall, the data show that these three genes, central to immune function, are upregulated during the polarization from M0 to M1 (Figure 5A), and this is significantly altered by 1% E-liquids with flavors, especially cinnamon (Figure 5B–D).

### 3.5. Cytokine Production

**Figure 6:**
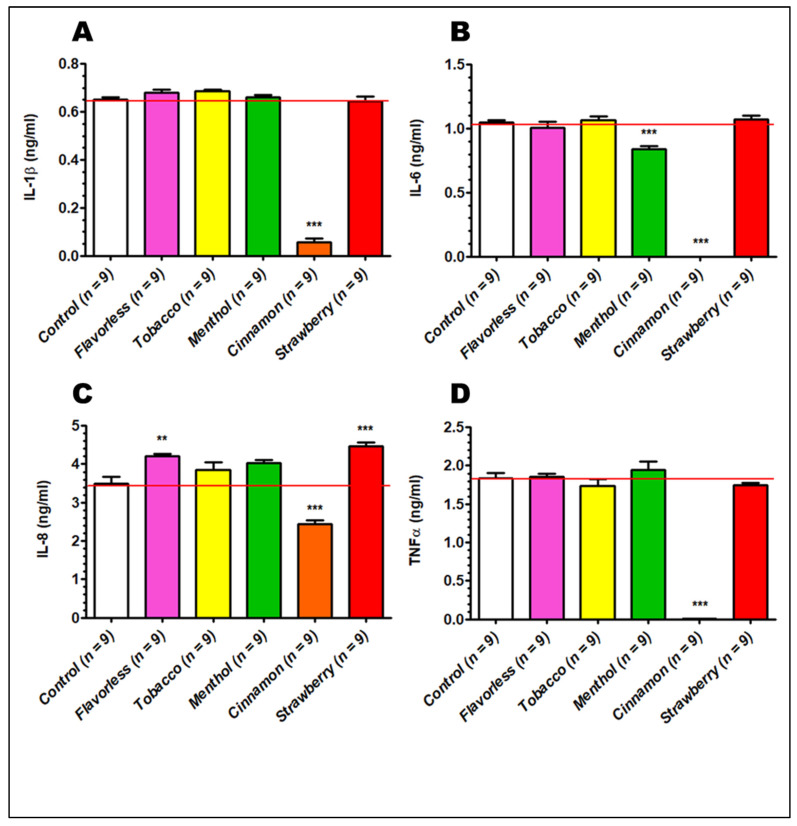
Cytokine profile 24 h after polarization from M0 to M1 macrophages with and without E-liquids ± flavors. Levels of IL-1β (**A**), IL-6 (**B**), IL-8 (**C**) and TNFα (**D**) were measured via ELISA and compared to untreated controls. Each bar represents the mean ± SEM; *n* = 6 to 12. ** = *p* < 0.01, *** = *p* < 0.001. Red lines indicate the control levels of gene expression.

Cytokine profiles were evaluated via ELISAs on the cell culture supernatants 24 h after polarization from M0 to M1 macrophages with and without 1% E-liquid ± flavors. Figure 6 shows the levels of IL-1β (Figure 6A), IL-6 (Figure 6B), IL-8 (Figure 6C) and TNFα (Figure 6D), where M1 control macrophages produce 0.65, 1.05, 3.49 and 1.83 ng/mL of each cytokine, respectively. In all cases, treatment with 1% cinnamon-flavored E-liquid significantly drops the production of all four cytokines (*p* < 0.001). In addition, treatment with 1% menthol-flavored E-liquid lowers the expression of IL-6 (*p* < 0.001). In contrast, flavorless and strawberry-flavored E-liquid treatments increase the expression of IL-8 (*p* < 0.01). Overall, 1% E-liquid treatments, especially cinnamon, significantly alter the cytokine profile 24 h after polarization from M0 to M1 macrophages.

## 4. Discussion

Governed by surrounding stimuli, the polarization from the M0 to the M1 macrophage subtype undergoes a specific set of sequential cellular and molecular events that are central to the inflammatory response in mucosa-associated lymphoid tissues (MALTs). This study reports the aberrations that occur in this polarization when the cells are exposed to E-liquids with flavors. The main parameters measured include morphology, cytotoxicity, gene expression of immune markers and cytokine production. Our results show that E-liquids, especially when flavored with cinnamon, cause a significant departure from the typical M1 immunobiology.

Most of the literature supports the results we observe in Figure 1, where undifferentiated THP-1 cells are susceptible to 1% cinnamon or menthol E-liquids, but during the transition from the M0 to the M1 subtype, there is no increase in LDH activity (Figure 4B). These results agree with Morris et al., reporting an increase in LDH in undifferentiated THP-1 cells after 24 h of cinnamon treatment, but not in M0 macrophages after cinnamon treatment [62]. A study by Ghosh et al. [63] shows that mint flavor induces significant levels of cytotoxicity on THP-1 cells. Muthumalage and coworkers also reported a cinnamon dose-dependent cytotoxic effect on Mono Mac 6 and U937 human monocytic cell lines [21]. Ma and coworkers (2021) [64] report that THP-1 cells exposed to 120 puffs of propylene glycol or glycerol result in pronounced cytotoxicity, with more than 50% cell death. Once differentiated with PMA, THP-1 cells become M0 macrophages (Figure 2). In vivo, macrophages are among the first responders to infections in the MALTs, which include a vast array of toxic compounds [65,66], making them resilient cells. This may explain the low levels of LDH activity on M0/M1 cells after E-liquid exposure (Figure 4B).

In our hands, M1 macrophages continue to elongate their pseudopodia, which is consistent with others [67,68,69]. Of importance, Clapp and coworkers exposed neutrophils to E-liquids with flavors and found that neutrophil morphology and formation of the neutrophil extracellular trap are decreased [47].

The gene expression of paramount immunological markers, including MHC class II, CD80 and TLR-4, is upregulated in M1 cells compared to M0 [30,33,70]. Our results in Figure 5A agree with these findings. Alarmingly, MHC class II and CD80 are significantly downregulated in the presence of E-liquid with cinnamon (Figure 5B,C). These results correlate well with other reports where the gene expression of ex vivo alveolar macrophages for canonical pro-inflammatory pathways from ECIG users is significantly reduced compared to those from non-vapers and non-smokers [71,72]. Of relevance, Saranyutanon et al. (2022) report a nicotine-dependent polarization to the M2 subtype [73]. Therefore, exposure to E-liquids hinders macrophage immune functions, including interactions with T cells.

Cytokine production is significantly reduced by cinnamon-flavored E-liquid (Figure 6), which agrees with most of the related literature. For example, Morris et al. also evaluated the levels of IL-1 β, IL-6, IL-8 and TNF α in M0 macrophages after 24 h treatments, with cinnamon and all cytokines being severely decreased [62]. In addition, IL-8 is also reduced in ex vivo alveolar macrophages from vapers [71]. Similarly, neutrophil IL-8 production is significantly decreased by 1% cinnamon E-liquid, among other flavors [47]. In contrast, Ma et al. (2021) showed that THP-1-derived M0 cells exposed to 480 puffs of flavorless aerosol without nicotine produce high levels of IL-1 β and TNF α [64]. As a matter of speculation, it is plausible that the experimental design, specifically exposing cells to E-liquids vs. aerosols, could be the main driver of the contrasting results.

Based on the macrophage morphology (Figure 3), reduced expression of MHC class II, CD80 (Figure 5) and cytokine profile (Figure 6), exposure to cinnamon-flavored E-liquid seems to abolish M0 polarization to M1. Since LDH activity is unaffected by cinnamon treatment (Figure 4B), these results could suggest an apoptotic process, but our data indicate otherwise. Light microscopy reveals that M1 polarization in the presence of cinnamon E-liquid for 72 h renders cells with a round morphology without presenting pseudopodia, cell density is unchanged, and there are no visible apoptotic bodies (Figure 3). In support of this claim, Shields and co-workers (2023) report that lung macrophages from ECIG users are 50% M0 and about 35% M1 cells [72]. Similarly, M0 cells for 21 days induce differences in cellular morphology, ranging from round to elongated and a “fried egg” appearance [74], with no claims to cell death. As a matter of speculation, it is possible that the cinnamon-treated cells in our study are still alive but have lost their ability to respond to stimuli that would promote their polarization to M1.

This study shows that E-liquid flavors, especially cinnamon, affect the polarization of M0 to M1 macrophages; however, there are several limitations that need to be considered. For example, this is an in vitro study. Second, this study includes a limited number of flavors. The flavors chosen are commonly found in the scientific literature, and because the list of possible flavors is extensive [8], we decided to use a few common representative flavors. Also, the dose used in this study was limited to 1% E-liquid. Furthermore, different doses or exposure times could have yielded more or less pronounced alterations in some of the other flavors tested. Although 72 h of exposure is unrealistic in terms of a true vaping session, this study illustrates the potential outcomes of this toxic exposure on macrophage immunobiology. Lastly, we only used THP-1-derived macrophages and not ex vivo human monocytes or any other cell line.

## 5. Conclusions

Overall, our results suggest that E-liquids, especially cinnamon, significantly alter or undermine polarization of M0 to M1 macrophages. This can be seen by alterations in the morphology, and expression levels of MHC class II, CD80 and TLR-4, as well as in the production of pro-inflammatory cytokines IL-1β, IL-6, IL-8 and TNFα. Such alterations could lead to adverse consequences in the innate immune response of the mucosal epithelium in the mouth and airways. These findings support previously reported ECIG-related irritation and symptoms associated with electronic cigarette and vaping-associated lung injury [75].

## Figures and Tables

**Table 1:** List of abbreviations in the order they appear in the article.

Abbreviations	Complete Terms
ECIG	Electronic cigarette
E-Liquid	ECIG liquid
PG	Propylene glycol
VG	Vegetable glycerin or glycerol
IL-	Interleukin-1β, 6, 8, 12, etc.
M1	Pro-inflammatory activated macrophages derived from M0 macrophages
M2	Alternatively activated macrophages associated with anti-inflammatory responses; derived from M0 macrophages
M0	Naïve macrophages derived from blood stream monocytes (THP-1 cells)
LPS	Lipopolysaccharide
THP-1	Tohoku Hospital Pediatrics-1; can differentiate into M0 macrophages
INF-γ	Interferon-gamma
TNF-α	Tumor necrosis factor-alpha
GM-CSF	Granulocyte-monocyte colony-stimulating factor
MHC	Major histocompatibility complex
CD80	Cluster of differentiation 80
TLR-4	Toll-like receptor-4
RPMI	Roswell Park Memorial Institute media
PMA	Phorbol 12-myristate 13-acetate
hrIFN-γ	Human recombinant interferon-gamma
LDH	Lactate dehydrogenase
HLA DR	Human Leukocyte Antigen DR isotype
SEM	Standard error of the mean
SD	Standard deviation
ANOVA	Analysis of variance
MALT	Mucosa-associated lymphoid tissue

## Data Availability

The data presented in this study are available on request from the corresponding author due to the large data sets used for each experiment. Please contact Dr. Cuadra if interested.
